# The role of EZH2 in overall survival of colorectal cancer: a meta-analysis

**DOI:** 10.1038/s41598-017-13670-z

**Published:** 2017-10-23

**Authors:** Laura Vilorio-Marqués, Vicente Martín, Cristina Diez-Tascón, María Francisca González-Sevilla, Tania Fernández-Villa, Emiliano Honrado, Veronica Davila-Batista, Antonio J. Molina

**Affiliations:** 1 GIIGAS: Grupo de Investigación en Interacción Gen-Ambiente-Salud, Dpt of Biomedical Sciences, Area of Preventive Medicine and Public Health, Instituto de Biomedicina (IBIOMED), University of León, Leon, Spain; 2 CIBERESP, CIBER de Epidemiología y Salud Pública, Madrid, Spain; 3 Banco de Tumores, Servicio de Anatomía Patológica, Complejo Asistencial Universitario de León, Leon, Spain; 4 GIIGAS: Grupo en interacción Gen-Ambiente-Salud, Dpt of Biomedical Sciences, Area of Physiology, University of León, Leon, Spain; 5 Servicio de Anatomía Patológica, Complejo Asistencial Universitario de León, Leon, Spain

## Abstract

Enhancer of zeste homolog 2 (EZH2) is the catalitic subunit of polycomb repressive complex 2 and mediates gene silencing. EZH2 is overexpressed in many cancers and correlates with poor prognosis. The role of the gene EZH2 in colorectal cancer survival is uncertainly, the aim of this study is clear this relationship. Relevant literaure was searched from electronic databases. A meta-analysis was performed with elegible studies which quantitatively evaluated the relationship between EZH2 overexpression and survival of patients with colorectal cancer. Survival data were aggregated and quantitatively analyzed. We performed a meta-analysis of 8 studies (n = 1059 patients) that evaluated the correlation between EZH2 overexpression and survival in patients with colorectal cancer. Combined hazard ratios suggested that EZH2 overexpression was associated with better prognosis of overall survival (OS) HR(hazard ratio) = 0.61 95% CI (0.38–0.84) We performed bias analysis according Egger and Begg,s test and we did not find publication bias. EZH2 overexpression indicates a better prognosis for colorectal cancer.

## Introduction

Colorectal cancer (CRC) is one of the main causes of death in industrialized countries, coming in third place for incidence and fourth for mortality in the world as a whole^1^.

Colorectal cancer develops from the progressive accumulation of molecular events, like somatic mutations in oncogenes, or epigenetic mechanisms such as methylation of DNA or post-transcriptional modification of histones^2^. Over recent years, various studies have focused on the discovery of the molecular changes that participate in the process of tumour development, with the aim of finding biomarkers potentially useful in predicting survival or directing therapeutic strategies^3^.

One of the mechanisms that regulate histone epigenetic modification is mediated by the polycomb repressive complexes (PcG). PcG are epigenetic modifiers that promote gene repression through modification and compaction of chromatin. Two major complexes, designated as PRC1 and PRC2, perform different functions in cells related to gene silencing^4,5^. PRC1 includes the sub-units Bmi1, Ring1b, CBX4 and PHC, and induces mono-ubiquitination of the residue of lysine 119 from histone H2. PRC2 is formed by the protein EZH2, a catalytic sub-unit with a methyl-transeferase activity, and the sub-units SUZ12, EED and RbAp48, necessary for maintaining the integrity of the complex^6^.

It is believed that the EZH2 protein participates in the transcriptional repression of genes through various mechanisms, such as trimethylation of the residue of lysine 27 of histone H3 (H3mek27), or methylation of the CpG islands It also operates as a platform recruiting other enzymes involved in gene silencing, like histone de-acetylases (HDACs)^7,8^ and methyltransferases (DNMT1, DNMT3A, and DNMT3b)^9^.

In cancer, EZH2 promotes cell proliferation, invasion, apoptosis, angiogenesis and metastasis, according to the findings of *in vitro* studies on different cell lines^10–13^. Moreover, it has been found over-expressed in the tumour tissues of numerous neoplasias affecting the prostate^7,14^, breast^15^, bladder^16^, ovaries^17^, small-cell^18^ and non-small-cell lung cancer^19^, brain tumours^20^, kidney cancer^21^, gastric cancer^22^, and cancer of the oesophagus^23^, pancreas^24^, or melanoma^25^.

A good number of studies suggest that the over-expression of EZH2 may have a prognostic value in some types of cancer, and it has been associated with a worse prognosis and survival rate in breast and prostate tumours^7,14,15,26^. As regards CRC, its role is less well known, and the mechanisms and routes in which it participates are not clear. A limited number of studies focus on the relationship between the expression de EZH2 and overall and disease free survival or with responses to treatment.

The present work undertakes a systematic review of studies that include the analysis of the expression of EZH2, and attempts to evaluate its influence over prognosis in CRC, through an analysis of the overall survival rates. Additionally, the methodological quality of the selected studies was assessed.

## Methods

### Search Strategy

A systematic review of the academic literature was performed, taking the expression of EZH2 as exposure variable and the survival from CRC as effect variable. The study was carried out in accordance with recommendations defined in the REMARKS (Reporting Recommendations for Tumour Marker Prognostic Studies) guide for meta-analyses and systematic reviews^27,28^.

Two researchers (Molina AJ and Vilorio-Marqués L) carried out independently searches for original works in Pubmed, Scopus and WOS (World of Science) among all material published up to August 2016, using the terms “EZH2” or “PCR2” and “Colorectal” or “Colon” and “Cancer” or “tumour” or “Neoplasm” or “Carcinoma”. A second phase incorporated a root search in respect of the papers included in the study, with the aim of detecting pieces of work that had not been netted in the first search.

### Selection of Papers

The systematic review included all those articles fulfilling the following criteria: studies done on sets of cases, cases and controls or cohorts of patients histologically diagnosed with CRC, studies determining the expression of EZH2 in human tissues by means of immunohistochemical techniques (IHC) or real-time quantitative polymerase chain reaction (q-PCR). The studies had to contain sufficient information for an estimate of Hazard Ratio (HR) relative to Overall Survival (OS), Disease-Free Survival (DFS), or both, with a confidence interval of 95%.

The review excluded *in vitro* or *ex vivo* studies, letters, narrative reviews, conferences summaries, or works related to other pathologies or neoplasias. The initial search was undertaken with no limitations as to language, but full paper reading and later assessment were only completed in works published in English.

### Data Collection

Molina AJ and Vilorio-Marqués L reviewed all the articles independently, checking their titles and abstracts, and gathered data from eligible studies. Disagreements were resolved through debate and consensus with a third researcher, Martín V.

The information collected for each study was: name of the first author, name of the journal, year of publication, type of study, number of cases, analysis of EZH2 expression, method employed (IHC and/or qPCR) data on follow-up, overall survival and disease-free survival (OS and DFS). The data selected for the research is summarized in Table 1.

### Methodological Assessment

In order to evaluate the methods used in the studies, three researchers, (Diez-Tascón C, Martín V and Sevilla F), read each publication independently and scored all of them for methodology and reproducibility in accordance with the REMARKS guidelines. In addition, a second scoring system established in a previous work, the “European Lung Cancer Working Party quality scale for biological prognostic factors for lung cancer” was used^29^. Four main categories were assessed, with several items per category: scientific design, methods used in the laboratory, generability and analysis of results. Each item was scored as follows: 2 points if well defined, 1 point if unclear or incomplete, and 0 points if undefined. According to the criteria proposed by Chen *et al*.^30^, a work was considered of high methodological quality if scored above 80%^29,30^.

### Statistical Analysis

Values for the hazard ratio (HR) and confidence intervals were taken directly from the articles when supplied by the authors, otherwise they were estimated through visual inspection of the Kaplan-Meier survival graphs. Survival data and time to events were extracted from the graphs, and the rate of data censored during follow-up was assumed constant^31^.

A meta-analysis was performed using the random effects model of DerSimonian and Laird^32^ to estimate the role of over-expression of EZH2 in survival from CRC, assuming a worse survival when HR > 1 The horizontal lines indicate confidence intervals of 95% and the boxes representing the estimates for HR are proportional to the weighting for the study. Moreover, to assess differences depending on the technique for detecting overexpression, meta-analysis were performed separately for q-PCR and IHC studies.

Additionally, the heterogeneity of the information (I^2^) was assessed, according to the Mantel Haenszel model. The I^2^ value measures the degree of inconsistency or incompatibility between studies, so higher values for I^2^ indicate greater heterogeneity^30^. An analysis of publication bias was carried out with the methods proposed by Egger *et al*.^33^ and Begg & Mazumdar^34^. For all these analyses, the statistical package Stata 13 was used.

## Results

### Search Procedure and Identification of Relevant Studies

**Figure 1:**
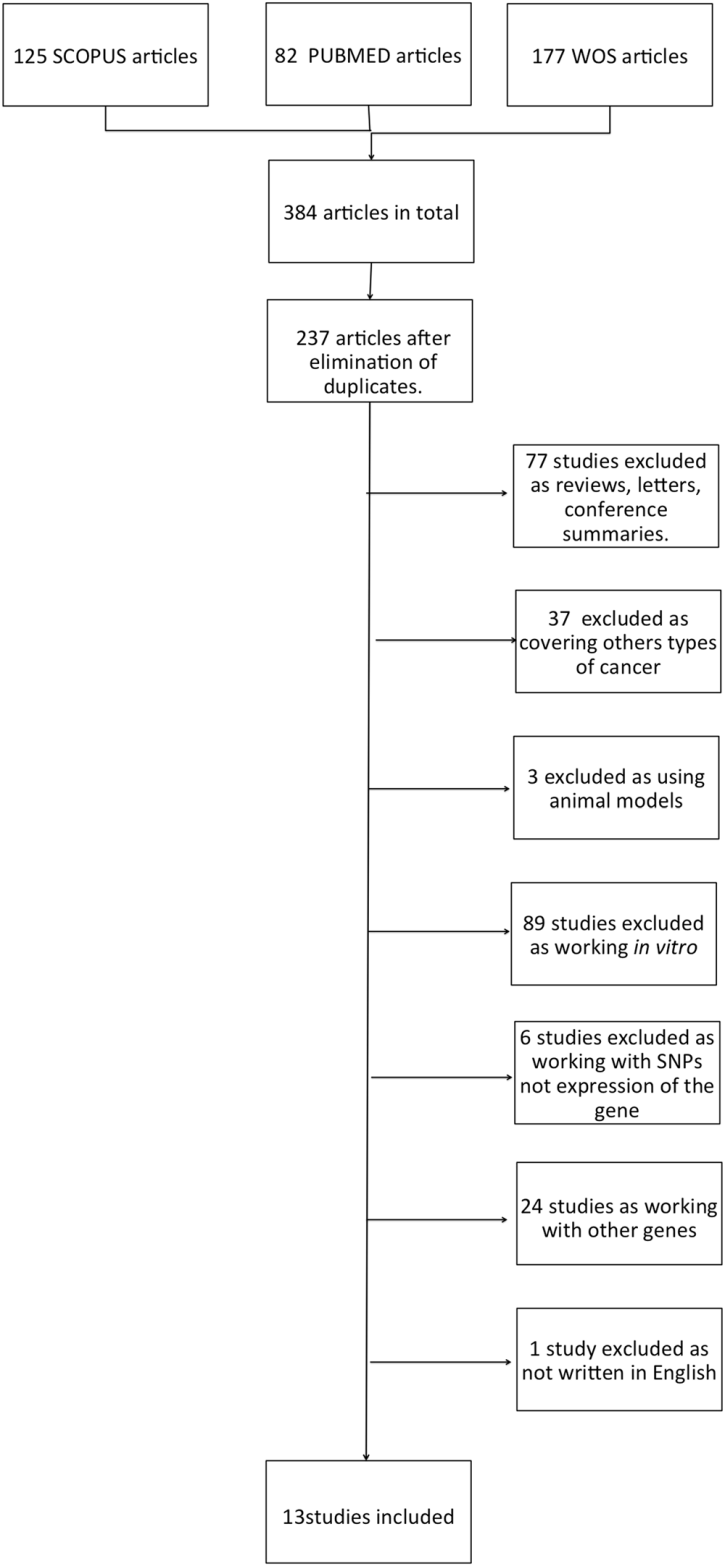
Flowchart for the Systematic Review.

During the initial search, we identified 384 articles from PubMed, Scopus and Web of Science databases. Of these, 134 duplicated works were eliminated. Subsequently, a first reading of the abstracts allowed to identify those studies that did not meet the defined selection criteria In brief, a total of 237 studies were excluded. Of those, 77 publications were narrative reviews, letters to the editor or conference summaries. 89 articles involved exclusively *in vitro* studies, 3 covered experimental work with a mouse model, 37 concentrated on other pathologies (malignant neoplasias other than CRC) 24 articles investigated other genes and 6 articles analysed polymorphisms (genetic variations) and not the expression of the gene and 1 study was in Chinese language. Finally, 13 studies resulted eligible for further assessment (Fig. 1).

The 13 fully reviewed articles were published in the period from 2005 to 2016. Of these, 6 studies were from Japan, 3 from China, 2 from the Netherlands, 1 from Norway and 1 from Greece. At this point 3 articles were further excluded after full reading, since they focused on the expression of EZH2 versus other markers (Long Non Coding RNA and mi-RNA)^35,36^, and treatments with statins^37^ rather than on the association between the expression of EZH2 and survival parameters.

Hence, ten studies resulted finally eligible for the systematic review. Assessment of their methodological quality showed compliance values ranging from 35.7% to 90.9%, with more than half scoring below 50% for quality and just two articles exceeding a quality threshold of 80% (Table 1). The studies looked at different measures for survival, studying OS (8 articles), DFS or RFS (3 articles) (Table 2). Of the ten studies, in six of them the authors found that over-expression of EZH2 tend to be an indicator of poor prognosis for colorectal cancer survival^38–43^, but only one of them reports statistically significant results^38^. The four remaining studies found instead that over-expression of EZH2 would be a good prognostic factor^44–47^, with two of them reporting statistically significant results^45,47^.

**Table 1:** Characteristics of Studies Eligible for the Systematic Review.

**Name of first author**	**Journal publishing study**	**Year of publication**	**Type of study**	**Method of detecting expression of EZH2**	**Number of cases**	**Survival measure**	**Methodology score**	**Included in study**
Kurihara H	Oncotarget	2016	Cohort study	IHC	310	OS	90.90	YES
Liu YL	Journal of Cancer Research and Clinical Oncology	2014	Case study	q-PCR	82	DFS	40.90	YES
Benard A	Public Library of Science One	2014	Cohort study	IHC	247	OS/DFS/RFS	90.90	YES
Jinushi T	Cancer Medicine	2014	Case study	-------	71	-------	-------	NO
Ishikawa S	International Journal of Cancer	2013	Case study	-------	742	-------	-------	NO
Tamagawa H	European Journal of Surgical Oncology.	2013	Case study	IHC	61	OS	35.71	YES
Lin Y	The Journal of Pathology	2013	Case study	IHC	129	OS	43.18	YES
Takawa M	Cancer Science	2011	Case study	IHC	172	OS	68.18	YES
Kogo R	Cancer Research	2011	Case study	-------	100	-------	-------	NO
Kodach LL	Carcinogenesis	2010	Case study	IHC	72	OS	40.90	YES
Wang CG	World Journal of Gastroenterology	2010	Case study	IHC	119	DFS	63.63	YES
Fluge	British Journal of Cancer	2009	Cohort study	IHC	409	RFS	84.09	YES
Mimori K	European Journal of Surgical Oncology	2005	Case study	q-PCR	61	OS	35.71	YES

**Table 2:** Details of the Studies Included in the Systematic Review. (T = length of time monitored; OS_C = raw overall survival rate; OS_A = adjusted overall survival rate; DFS_C = raw disease-free survival rate; DFS_A = adjusted disease-free survival rate; RFS_C = raw recurrence-free survival rate; RFS_A = adjusted recurrence-free survival rate).

**AUTHOR**	**YEAR**	**Technique**	**Cutoff over-expression**	**N**	**T(YEARS)**	**OS_C 95% CI**	**OS_A 95% CI**	**DFS_C/RFS_C 95% CI**	**DFS_A/ RFS_A 95% CI**
Kurihara_2016	2016	IHC	− → < 80% Mean nuclear positivity + → ≥ 80% Mean nuclear positivity	301	4.4	**0**.**48**(0.29–0.77)			
Benard_2014	2014	IHC	− → ≤ median % of nuclear positivity + → > median % of nuclear positivity	247	8.6	**0**.**74**(0.54–1.03)	**0**.**84**(0.60–1.18)	**0**.**64**(0.42–0.99)	**0**.**67**(0.43–1.05)
Liu_2014	2014	q-PCR	− → ≤ median tumor/normal ratio + → > median tumor/normal ratio	82	3	—	—	**4**.**18**(2.08–8.36)	**2**.**52**(1.10–5.73)
Tamagawa_2013	2013	IHC	− → ≤ median value of H-score EZH2 (0–300) + → > median value of H-score EZH2 (0–300)	54	8	**1**.**09**(0.50–2.44)	—	—	—
Lin_2013	2013	IHC	Index value calculated as a product of the grades of the extent and intensity of staining:− → Grades 0–3 + → Grades 4–12	33	5.8	**5**.**07**(1.51–16.7)	—	—	—
Takawa_2011	2011	IHC	− → No appreciable staining in tumor cells + → Brown staining appreciable in the nucleus of tumor cells	172	5	**0**.**39**(0.19–0.83)	**0**.**42**(0.18–0.97)	—	—
Kodach_2010	2010	IHC	− → < 70% positive tumor cells + → ≥ 70% positive tumor cells	72	6.6	**1**.**54** (0.29–8.38)	—	—	—
Wang_2010	2010	IHC	− → ≤ 30% positive tumor cells + → > 30% positive tumor cells	119	6.6	—	**3**.**21**(1.06–9.72)	—	—
Fluge_2009	2009	IHC	Index value calculated as a product of the grades of the extent and intensity of staining:− → Grades 0–3 + → Grades 4–9	409	7	—	—	**1**.**17**(0.46–2.98)	—
Mimori_2005	2005	q-PCR	− → ≤ median tumor/normal ratio + → > median tumor/normal ratio	61	8	**2**.**17**(0.93–5.07)	—	—	—

### Meta-Analysis

**Figure 2:**
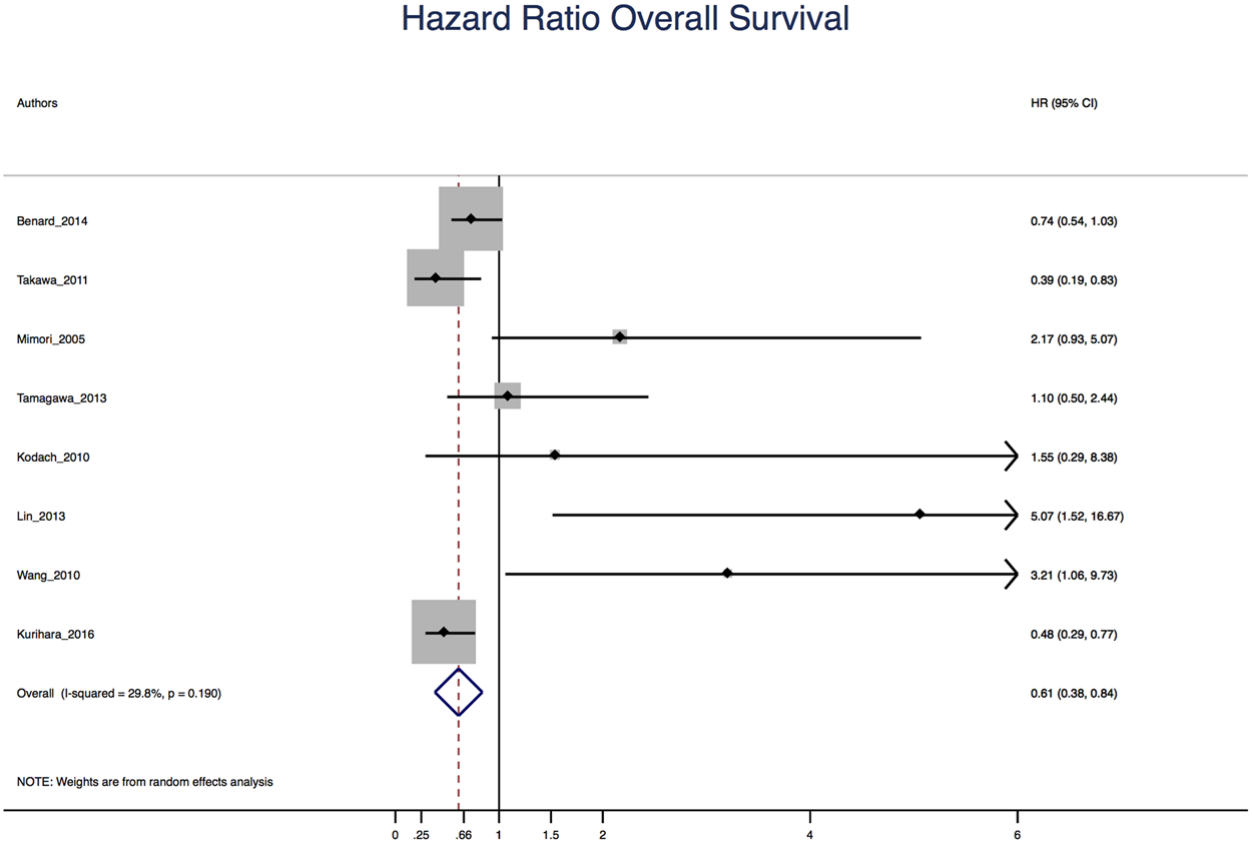
Plot for the Meta-Analysis of Raw Overall Survival (OS).

Eight studies that investigated overall survival were included in the meta-analysis. In five of them the HR was reported, while in the remaining three it was estimated from the Kaplan Meier survival graphs. The overall HR obtained was HR = 0.61 CI 95% (0.38–0.84) (Fig. 2). These data suggest that in CRC over-expression of EZH2 is a good prognosis factor for survival. In respect to the heterogeneity of the studies the value for I^2^ was 29.8%, which is not statistically significant (p = 0.190). In addition, we analyzed and performed the meta analysis specifically for the seven studies using IHC obtaining an HR = 0.58 CI 95% (0.38–0.79).

### Analysis of Publication Bias

**Figure 3:**
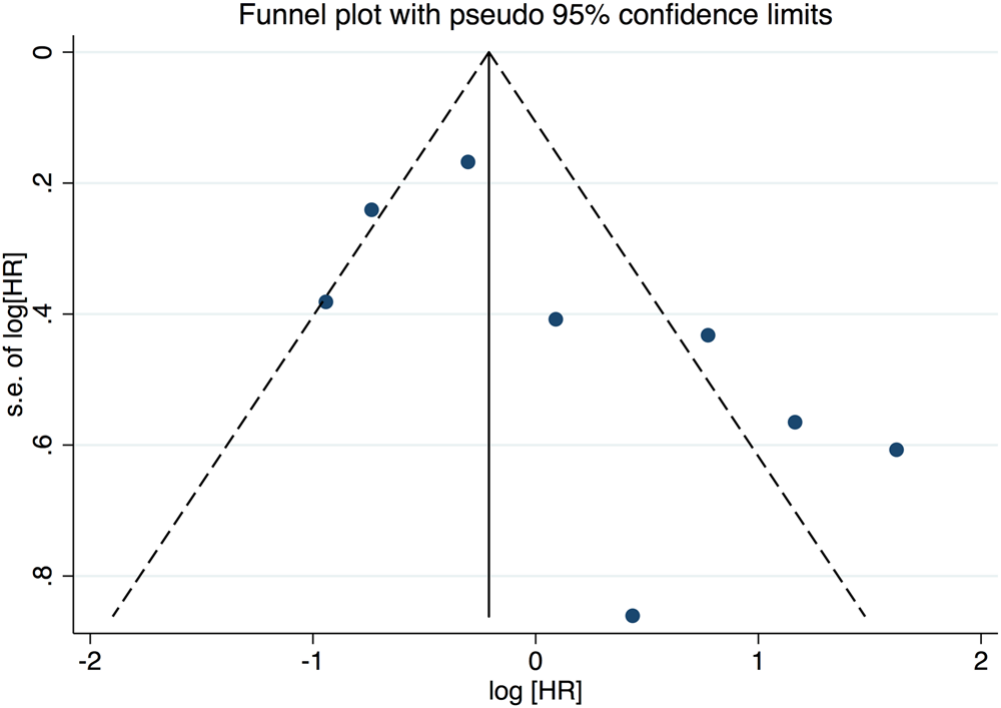
Funnel Plot for the Eight Studies Investigating the Role of EZH2 in Overall Survival from CRC.

Although the funnel plot (Fig. 3) showed some asymmetry, neither Egger’s test (p = 0.121) nor Begg’s test (p = 0.072) were statistically significant. These results suggest that there is no publication bias for the over-expression of EZH2 in survival from CRC.

## Discussion

The systematic review presented in this article identified ten studies evaluating survival from CRC related to the expression of EZH2 in tumour tissue, with OS as a measure of survival in eight of these works. A meta-analysis was performed by merging the eight studies and including a total of 1059 CRC patients. The combination of HR suggested that over-expression of EZH2, is associated with a better prognosis and a better OS with an overall HR = 0.61 CI 95% (0.38–0.84) this result was statistically significant.

These data are consistent with the values obtained by Wang *et al*.^48^ and Jiang *et al*.^49^, who in two recently published meta-analyses found values for overall HR = 0.91 CI 95% (0.63 to 1.19) and HR = 0.75 CI 95% (0.28 to 1.22) respectively for the relationship between the expression of EZH2 and survival from CRC. On the other hand, another previous meta-analysis^30^, which covered even more tumour types, that included just two articles for CRC with a total of 291 patients, reported an overall HR = 1.13 CI 95% (0.16 to 8.21), which is not in line with our results. In the present review, we have incorporated more articles referring to CRC that those consider by these previous meta-analysis.

This finding of a protective role of over-expression of EZH2 for CRC is noticeable, because in other solid tumors, such as prostate, breast and lung, the available data support an opposite effect than ours^8,26,50^. Moreover, it is known that EZH2 is overexpressed in tumour tissue as compared to healthy tissue^14,15,18,20,51^, and it has been suggested, and supported by convincing mechanicistic data, an oncogenic function of EZH2 related to PRC2 functioning that, through histone methylation, would lead to chromatin condensation repressing the expression of tumour suppressor genes^52^. This process would be associated with a worse prognosis and lower survival rate^4^.

Nevertheless, recent studies suggest that EZH2 is a dual-faced molecule which may act as transcripcional repressor, but also as an activator^53–55^. Post-translational modifications^56^, variations in its association with other PRC2 subunits, such as SUZ12 and EED^57^ and the existence of PRC2-independent activity of EZH2^58,59^, and the role of SNPs in their expression or functionality^60–62^ are some of the proposed reasons for this variability in the role of EZH2 in cancer survival. In addition, other studies have shown that a loss of the trimethylation state of H3k27 is related to lower survival rates in cancers of breast^63,64^, ovaries, pancreas^63^ and rectum^65^, which would be consistent with the results found in the present meta-analysis. Recently, Wassef & Margueron, suggests that taken into account its biological function as an important layer of gene regulation, is not surprising that a lower PRC2 function could be related to an oncogenic effect due the possible activation of genes that would otherwise remain silent^55^.

Furthermore, these findings would support the hypothesis that there exist methylation patterns of histones, genes and the wide genome so tumour genesis would be associated with hypomethylation in histones and non-coding genome together with hypermethylation at specific loci and in individual CpG islands of specific genes^66^.

To the best of our knowledge, the present study is the most comprehensive meta-analysis hitherto on the role of EZH2 in survival from colorectal cancer. We performed this systematic review and meta-analysis to yield summary statistics by including more recent related studies and by generally using a more comprehensive search strategy. Screening, study selection, and quality assessment were performed. We also explored heterogeneity and potential publication bias in accordance with published guidelines.

Nevertheless, the limitations of this work include the number of articles included, which restricts interpretation using the methods of Egger and Begg, despite the fact that no publication bias of statistical significance were found^67^. It should not be forgotten, either that all the publications considered were written in English, and the tendency in the academic world is to publish the most striking results in international journals whereas local journals and grey literature report not so impactful findings.

Moreover, studies may differ with regard to the baseline characteristics of the patients included age, disease stage, duration of follow up, and also in the use of different techniques to assess the expression of EZH2 and the absence of a standarized cut-off to consider overexpression in each of these techniques. All these variability sources could suppose limitations for comparison, as has been previously reported in other meta-analysis^30,49^.

Another potential source of bias relates to the method of extrapolating HR and the 95% confidence interval, because these were calculated from data provided in articles, or else they were extrapolated from survival curves, when not reported by authors, which necessarily involved assumptions being made about any censoring process.

In conclusion, the present meta-analysis suggest a protective role for over-expression of EZH2 in survival from CRC. Nevertheless, the paucity of available studies limits its potential application as a prognostic biomarker. Therefore, further studies, both retrospective and prospective, are warranted in order to explore the expression of EZH2 as a biomarker for survival and to clarify the molecular mechanisms that involve EZH2 in colorectal cancer.
